# Relevância das redes sociais na mobilização social para o enfrentamento de arboviroses no Município de Betim, Minas Gerais, Brasil 

**DOI:** 10.1590/0102-311XPT214722

**Published:** 2023-07-17

**Authors:** Paloma Coelho, Júlia Vargas Batista, Zélia Profeta

**Affiliations:** 1 Instituto René Rachou, Fundação Oswaldo Cruz, Belo Horizonte, Minas Gerais, Brasil.; 2 Instituto de Filosofia e Ciências Humanas, Universidade Estadual de Campinas, Campinas, Brasil.

**Keywords:** Redes Sociais, Capital Social, Participação da Comunidade, Participação Social, Vigilância em Saúde Pública, Social Networks, Social Capital, Community Participation, Social Participation, Public Health Surveillance, Redes Sociales, Capital Social, Participación de la Comunidad, Participación Social, Vigilancia en la Salud Pública

## Abstract

Este artigo consiste em uma análise das redes sociais dos moradores de uma comunidade em Betim (Minas Gerais, Brasil), visando compreender como elas podem ser utilizadas nas estratégias de mobilização social para o enfrentamento da dengue, zika e chikungunya no território. Utilizou-se o método da trajetória de vida para analisar os eventos e os condicionantes sociais da formação, manutenção e ruptura dessas redes, a qualidade e intensidade dos vínculos, as características do capital social e sua variação ao longo da trajetória dos indivíduos. A compreensão da estrutura das redes evidencia alguns aspectos importantes para a elaboração de novas estratégias de mobilização social no âmbito da proposta de vigilância em saúde a ser implementada no local. Na trajetória dos entrevistados, a vizinhança se mostrou como importante rede de reciprocidade e de provisão de recursos no cotidiano, dada a proximidade física e a duração das relações. Além disso, as redes religiosas têm presença significativa no cotidiano dos moradores, sendo fortemente ancoradas na prestação de “ajuda” social e pautada por valores solidários. Acredita-se que os comitês populares possam estimular essas redes, sobretudo as religiosas, a utilizarem seu repertório cultural e simbólico para trabalhar questões de interesse dos bairros, como promoção da saúde, construção de territórios saudáveis e sustentáveis, desenvolvimento local, geração de renda, melhoria da infraestrutura e preservação ambiental.

## Abstract (en)

This article consists of an analysis of the social networks of the residents of a community in Betim, in the State of Minas Gerais, Brazil, to understand how they can be used in social mobilization strategies against dengue, zika, and chikungunya within the territory. The life trajectory method was used to analyze the events and social conditioning factors of the formation, maintenance, and rupture of these networks, the quality and intensity of the bonds, the characteristics of social capital, and its variation along the trajectory of individuals. The understanding of the structure of the networks highlights some important aspects for the elaboration of new social mobilization strategies within the scope of the health surveillance proposal to be implemented locally. Within the trajectory of the interviewees, the neighborhood was revealed as an important network of reciprocity and provision of resources in daily life, considering the physical proximity and the duration of the relationships. Additionally, religious networks have a significant presence in the daily lives of residents, being strongly anchored in the provision of social “assistance” guided by solidarity values. We believe that the popular committees can stimulate these networks, especially the religious ones, to use their cultural and symbolic repertoire to work on issues of interest to the neighborhoods, such as the promotion of health, promotion of healthy and sustainable territories, local development and income generation, infrastructure improvement, and environmental preservation.

## Resumen

Este artículo consiste en un análisis de las redes sociales de los habitantes de una comunidad en Betim, Minas Gerais, Brasil, buscando comprender cómo pueden ser utilizadas en las estrategias de movilización social para el enfrentamiento del dengue, virus del zika y chikungunya en el territorio. Se utilizó el método de la trayectoria de vida para analizar los eventos y los condicionantes sociales de la formación, mantenimiento y ruptura de esas redes, la calidad e intensidad de los vínculos, las características del capital social y su variación a lo largo de la trayectoria de las personas. La comprensión de la estructura de las redes evidencia algunos aspectos importantes para la elaboración de nuevas estrategias de movilización social en el ámbito de la propuesta de vigilancia en salud a ser implementada en el lugar. El vecindario se mostró en la trayectoria de los entrevistados como importante red de reciprocidad y de provisión de recursos en el cotidiano, dada la proximidad física y la duración de las relaciones. Además de estas, las redes religiosas tienen una presencia significativa en la vida cotidiana de los residentes, estando fuertemente ancladas en la prestación de “ayuda” social y pautada por valores solidarios. Se cree que los comités populares pueden estimular esas redes, sobre todo las religiosas, a utilizar su repertorio cultural y simbólico para trabajar en temas de interés de los barrios, como la promoción de la salud, la promoción de territorios saludables y sostenibles, desarrollo local y la generación de ingresos, la mejora de la infraestructura y preservación la ambiental.

## Introdução

Em 2015 e 2016, o Brasil vivenciou a tríplice epidemia de dengue, zika e chikungunya, considerada uma das maiores tragédias de saúde pública do país ^1^, levando o Ministério da Saúde a declarar emergência em saúde pública de importância nacional. A recorrência da epidemia de dengue no Brasil evidencia o baixo êxito na redução da circulação dos vírus, apesar dos esforços empregados desde os anos 1980 para a erradicação do *Aedes aegypti*^2^.

A dificuldade de mobilização social para o enfrentamento das epidemias remete à necessidade de refletir sobre estratégias pedagógicas, de comunicação e de participação civil e coletiva capazes de estimular o engajamento da população para questões próprias do seu território. Envolver as comunidades nas ações de vigilância em saúde é uma estratégia preconizada pela Organização Mundial da Saúde (OMS) em âmbito global, que reafirma a importância de fomentar um processo ativo de participação comunitária para identificação, notificação, resposta e monitoramento de eventos de saúde ^3^.

Partindo desses desafios, um grupo de pesquisadoras(es) vem desenvolvendo, desde 2016, a *Proposta de Vigilância em Saúde, de Base Territorial, Visando ao Fortalecimento da Mobilização Social para o Enfrentamento de Dengue, Zika, Chikungunya e Controle do* Aedes aegypti *em Minas Gerais* (também chamada de *Vamo Junto? Enfrentando a Dengue, Zika e Chikungunya*). O projeto consiste na formação de comitês populares para definir e implementar estratégias participativas para reconhecimento, análise e discussão sobre o território. A iniciativa visa à elaboração de um diagnóstico da situação de saúde e das condições de vida que contribua para o planejamento de propostas de mobilização social para o controle do *Aedes aegypti* e para a criação de ambientes favoráveis à saúde. Os comitês têm um(a) coordenador(a), escolhido pelos integrantes, e toda a atividade é realizada via plataforma *online*, criada para o desenvolvimento da proposta. Nela, os participantes encontram informações sobre as doenças e orientações sobre as atividades a serem realizadas, intermediadas por tutores e pela equipe de pesquisa. O trabalho dos comitês consiste em realizar um diagnóstico do território, elaborar um planejamento de ações e acompanhar sua implementação ^4^.

A primeira fase do projeto evidenciou alguns desafios para a participação e para o envolvimento das comunidades locais. Sediados em escolas da rede pública, os comitês foram, em sua maioria, formados por alunos, professores e demais funcionários. Consequentemente, a participação ficou restrita ao ambiente e ao calendário escolar, gerando limitações tanto ao alcance quanto à sustentabilidade do projeto.

Com o intuito de estimular o protagonismo das comunidades para além dos muros das escolas, propôs-se repensar as estratégias de mobilização social para ampliar o alcance do projeto por meio da inclusão de outros grupos e esferas sociais existentes nos territórios. Para isso, iniciou-se uma pesquisa que visa analisar as redes sociais de uma comunidade em Betim (Minas Gerais, Brasil), município selecionado para o desenvolvimento da segunda etapa do projeto, a fim de identificar as esferas sociais mais atuantes e com maior concentração de capital social que possam contribuir para o aumento da abrangência do trabalho dos comitês populares. As redes sociais locais são fundamentais por serem geradoras de círculos de reciprocidade e confiança entre os moradores de um território. Compreender suas características, dinâmicas e usos no cotidiano permitirá utilizá-las na mobilização social como canais de difusão de informações, de conhecimento e de outros recursos que as potencializem para construir formas de participação em que a população, como protagonista, possa vocalizar suas necessidades, propor soluções e refletir sobre suas práticas de saúde. Embora a literatura sobre redes sociais aponte para a maior presença de redes primárias, pautadas por laços fortes, com elevado grau de homofilia (tendência de indivíduos estabelecerem vínculos com outros que têm atributos comuns aos seus ^5^) e de localismo (característica de redes sociais formadas predominantemente por contatos localizados na mesma região ou lugar de moradia ^5^) nas periferias urbanas, resultando em baixo nível de capital social ^5^, nossa hipótese é de que haja nessas comunidades instâncias sociais atuantes, que geram círculos de reciprocidade e de confiança. Essas redes operariam como agentes de produção de solidariedade no território, mas não necessariamente estariam articuladas às esferas políticas, nem passariam por vias “formais” dos espaços de elocução pública.

Este artigo visa analisar as redes sociais de um grupo de moradores dessa comunidade, buscando compreender suas características, identificar os principais recursos que circulam nessas redes e as esferas sociais em que estão concentrados, a intensidade e os fatores que favorecem a manutenção e ruptura dos vínculos. A partir disso, busca-se refletir sobre como essas redes podem ser utilizadas nas estratégias de mobilização social para o enfrentamento da dengue, zika e chikungunya no território estudado.

## Redes sociais: conceitos e aplicações

A noção de rede social é relevante para os estudos de comunidades porque seu foco de análise é a relação social, a partir da compreensão dos padrões de conexão entre os sujeitos, e não dos atores individuais com seus interesses e atributos. Trata-se da constituição dos laços de sociabilidade entre os indivíduos e suas afiliações a grupos. O estudo das redes sociais corresponde à reconstrução desses processos interativos em determinado contexto, ou seja, das conexões interpessoais que os sujeitos constroem em seu cotidiano ^6^. Assim, possibilita a compreensão da estrutura de sociabilidade no interior de cada campo social em que os indivíduos, interligados por diversos círculos sociais, se inserem, sustentados pelos vínculos de pertencimento entre seus membros ^7^.

“...*Esse fenômeno pode ser analisado a partir do indivíduo e da constituição de seus processos interativos - amizade, parentesco, relações de trabalho, ligações territoriais etc. -, em que os padrões de sociabilidade são pensados em relação à disposição desses atores nas redes sociais. As instituições que interagem com essas redes podem ser dimensionadas enquanto agentes potencializadores ou desagregadores*” ^6^ (p. 54).

Esse instrumento analítico permite apreender as características e a estrutura das redes, além da maneira como elas são acionadas para a obtenção de recursos (materiais e imateriais). Permite, ainda, compreender de que forma a inserção nas redes condiciona as oportunidades e os limites de acesso a esses bens.

Cada esfera de sociabilidade é caracterizada pelo tipo de laço social estabelecido nas redes. Define-se sociabilidade primária como aquela em que as relações são estabelecidas por meio de laços fortes, em que a relevância das interações está em seu caráter pessoal, como as que se constituem na família, na vizinhança e nas amizades. Sociabilidade secundária é o tipo de relação social regida pela impessoalidade, pautada por laços fracos, como a que se constrói nas esferas do mercado, dos órgãos públicos, das instituições científicas, das associações civis, das organizações não governamentais (ONG), entre outras ^6^^,^^8^.

Na área da saúde, o conceito de redes sociais tem sido bastante útil para repensar as relações entre as políticas e instituições e as comunidades, havendo muitas abordagens teórico-metodológicas e de intervenção utilizadas. Bruno Fontes ^7^ propõe o conceito de “relés” como instrumento analítico para explicar, a partir das redes egocentradas (círculo de interações mais próximas de um indivíduo, formado por contatos e vínculos primários ^5^), os processos de mobilização subjacentes a um movimento social. Os relés sociais seriam as redes que produzem e geram a mobilização para novas redes, criando um canal que conecta os indivíduos a uma trama de novas sociabilidades. Juntamente, Martins & Fontes ^9^ discutem a ideia de uma rede de vigilância em saúde que incorpore o contínuo diálogo e a participação da comunidade, constituindo a principal responsável pelo controle das políticas públicas. As ações de intervenção baseadas na ideia de rede social pressupõem que a mobilização de recursos sociais locais seja essencial para o desenvolvimento de políticas sociais efetivas e descentralizadoras. Para isso, os autores ressaltam a necessidade de compreender como as redes sociais operam em cada contexto, identificando os tipos de redes predominantes, bem como seu impacto organizacional e político ^9^.

As redes de solidariedade são um importante instrumento de proteção, pois, além de oferecerem possibilidades de inserção social, estimulam a cidadania ao levar a sociedade civil à participação ativa no planejamento e na execução de ações locais. Tanto as redes como a noção de solidariedade são cruciais para se pensar em políticas sociais na contemporaneidade, especialmente em contextos em que se verifica uma considerável omissão do Estado no atendimento às demandas sociais, criando espaço para emergência de solidariedades locais e para a participação local ^8^.

## Metodologia

O estudo das redes sociais foi feito por meio do método da trajetória de vida, que consiste na relação entre as experiências de vida e o contexto social em que os indivíduos estão inseridos ^10^^,^^11^. Nesta pesquisa, o método foi utilizado para compreender as formas como os indivíduos se inserem nas redes sociais, criando e rompendo vínculos ao longo da vida, a partir da análise dos eventos e dos condicionantes sociais da criação, da manutenção e da perda de vínculos. A trajetória de vida permite, ainda, compreender a maneira como a inserção nessas redes favorece ou dificulta o acesso aos recursos, além das características do capital social e sua variação ao longo da trajetória dos indivíduos.

Os primeiros contatos com a comunidade foram feitos por meio do levantamento de lideranças locais realizado junto à administração da regional que abrange os bairros estudados e do levantamento *online* das redes existentes no território. A partir daí, foi utilizada a técnica “bola de neve”, em que cada entrevistado indicou alguém da sua rede. De novembro de 2020 a janeiro de 2021, foram feitas 30 entrevistas, abrangendo distintas faixas etárias, níveis de escolaridade, profissões, locais de moradia e formas de atuação nos bairros estudados. O número de entrevistas foi determinado pelo critério de saturação, quando se chegou a um ponto de repetição das informações que já não alterava a compreensão do fenômeno estudado.

Para a realização das entrevistas, foi elaborado um roteiro prévio com tópicos centrais, cujos temas - relacionados ao problema de pesquisa - foram abordados dando-se espaço para os entrevistados se expressarem da maneira mais livre possível. Foram feitas intervenções apenas para esclarecer melhor algum ponto ou explorar algum assunto relevante, técnica denominada “entrevista centrada no problema de pesquisa” ^12^. Os temas, com enfoque nas redes de sociabilidade, se centraram na infância e nas vivências no bairro de moradia, formação escolar/acadêmica, trajetória profissional, relações familiares, relações de vizinhança e comunidade, relações de amizade, participação em grupos e associações, além da relação e percepção sobre o bairro de moradia.

Os resultados não têm a pretensão de reduzir a complexidade das dinâmicas societárias do universo estudado, pois a análise diz respeito às relações sociais de uma parcela de moradores, não refletindo a totalidade das redes sociais dessa comunidade. Ainda assim, acredita-se que este estudo constitui um recorte relevante para se compreender parte dos mecanismos de solidariedade presentes naquele contexto.

Os nomes referidos nas entrevistas são fictícios para resguardar a identidade das(os) moradoras(es). Os procedimentos de pesquisa foram aprovados pelo Comitê de Ética em Pesquisa do Instituto René Rachou, Fundação Oswaldo Cruz, Minas Gerais (parecer nº 3.324.161).

## Contextualização do território

A comunidade estudada abrange três bairros de Betim, pertencentes à Regional Alterosas: Alterosas II, Cruzeiro do Sul e Duque de Caxias. Os três foram selecionados por serem os bairros da regional com a maior incidência de dengue, zika e chikungunya verificada nos últimos anos por meio dos levantamentos das autoridades municipais. O Município de Betim faz parte da Região Metropolitana de Belo Horizonte e é um dos principais polos industriais do estado. Em 2020, tinha cerca de 440 mil habitantes, com densidade populacional de 1.102,8 habitantes/km^2^. O Índice de Desenvolvimento Humano (IDH) é de 0,749 e o Produto Interno Bruto (PIB) *per capita* em 2020 foi de aproximadamente R$ 58.870,00 ^13^. A cidade é dividida em dez regiões administrativas, sendo a Regional Alterosas a mais populosa (aproximadamente 96 mil habitantes). A regional é composta por 29 bairros e sua população corresponde a mais de 22% do contingente populacional total do município ^14^.

Com base no *Censo Demográfico* de 2010, o bairro Alterosas II é o mais populoso dos três, com 19.536 habitantes. Nele, há 5.956 domicílios, com ocupação de 94% ^15^. O bairro Cruzeiro do Sul tem 6.243 habitantes, com 1.916 domicílios, 95% ocupados ^16^. Já Duque de Caxias é o menor e menos populoso dos três bairros, com população de 4.498 habitantes, 1.414 domicílios permanentes, 94% deles ocupados ^17^. Em média, a população dos três bairros é composta por 70% de moradores com faixa etária entre 15 e 64 anos.

## Resultados e discussão

Foram entrevistados 16 homens e 14 mulheres, com idade entre 20 e 70 anos; 15 deles com Ensino Superior completo (oito com Pós-graduação), 14 com Ensino Médio completo e um com Ensino Fundamental completo; 23 se declararam negros (pretos e pardos) e sete se declararam brancos; 11 têm renda familiar de um a três salários mínimos, nove têm renda familiar maior que três e menor ou igual a cinco salários mínimos, sete têm renda familiar maior que cinco e menor ou igual a 10 salários mínimos e três têm renda familiar maior que 10 salários mínimos; 13 são moradores, ex-moradores ou atuam no bairro Alterosas II, nove são moradores, ex-moradores ou atuam no bairro Duque de Caxias e oito são moradores, ex-moradores ou atuam no bairro Cruzeiro do Sul. Ainda, 27 entrevistados têm vínculo com um dos três bairros por mais de 10 anos.

As características dos bairros são importantes para compreender a conformação das redes sociais ao longo do tempo. A precariedade socioeconômica, aliada à carência de infraestrutura urbana adequada, contribuiu para a constituição de uma significativa rede de apoio social entre os primeiros moradores, marcada pela constante circulação de recursos (trocas) e pela forte coesão social. De modo geral, as relações de vizinhança podem ser assim tipificadas: no início da ocupação dos bairros, eram pouco numerosas, o que favoreceu a criação de vínculos estreitos entre os moradores. Praticamente todos relataram grande proximidade da vizinhança nessa época, com a presença de crianças brincando nas ruas, interações que se estendiam aos pais e às famílias, que frequentavam a casa uns dos outros; a vizinhança era vista como uma extensão da casa. O fato de os moradores serem migrantes de cidades do interior do estado contribuiu bastante para esse tipo de interação, já que muitos entrevistados afirmaram que a convivência entre os vizinhos lembrava muito as do interior. Pode-se dizer o mesmo das relações de vizinhança de favelas e de bairros periféricos da Região Metropolitana de Belo Horizonte, caracterizados pelos entrevistados como de muita proximidade, ajuda mútua e intimidade. Tal qual encontrado nas redes sociais estudadas por Fontes & Eichner ^6^, a territorialidade é um importante fator de integração social, em decorrência da identificação pelo compartilhamento de modos de vida e de aspectos culturais comuns.

Em contrapartida, as relações de vizinhança mais típicas de bairros de classe média ^5^ apareceram na trajetória de dois entrevistados: sem muita intimidade e proximidade. No caso de um deles, suas relações de vizinhança em Contagem se restringiam aos familiares, já que a família extensa morava em casas no mesmo lote. O mesmo acontece em Betim, onde ele diz não ter muito contato com os vizinhos. Leandro, pastor em uma igreja na região, percebe lá uma relação maior de comunhão entre os vizinhos do que no bairro em que mora, mais bem desenvolvido e urbanizado: “*até na congregação da igreja existe muito isso, entre pessoas que se conhecem e cresceram junto, né? Ainda existe isso, menos, mas existe. Até por ser um bairro mais humilde, entre aspas, ainda persiste essa questão de amizade, comunhão em alguns casos*”.

Contudo, a maioria relatou o enfraquecimento desses laços ao longo dos anos, em decorrência da urbanização, do aumento da violência e da chegada de novos moradores, vindos de outras cidades e até de outros países. Essa mudança alterou a intensidade dos vínculos - vizinhos deixam ou diminuem o costume de frequentar a casa e de participar da vida uns dos outros. No caso dos novos moradores, a relação se limita às interações ocasionais e impessoais, como o cumprimento rápido nas ruas. Alguns entrevistados ressaltaram o individualismo presente nas relações atuais. Para eles, há uma tendência dos vizinhos a se importar apenas consigo mesmos. Entretanto, mesmo com essas transformações, verificou-se a manutenção de fortes vínculos entre os moradores antigos, motivados por um sentimento de pertencimento e de identidade em relação ao bairro. Alguns, inclusive, se consideram como membros da família:

“*Na vizinhança, e a vizinhança que estou falando não era só vizinhança da frente, era do lado, era de baixo, da rua de cima, eram todos ao redor ali, e nós fomos crescendo como se fôssemos realmente um grupo familiar, além de sermos, lógico, vizinhos, parecíamos um grupo familiar, todo mundo conhecia todo mundo*” (Éder).

“*Nós somos muito próximos, nós somos muito amigos também, não é à toa que a* [nome da vizinha] *tem um filho e eu sou muito próximo dela. Pra você ter ideia, eu não sou irmão dela e o filho dela me chama de tio. Eu sou padrinho dele, entendeu?* (...) *Eu lembro, quando eu era pequeno, eu não lembro como foi, eu tinha um vizinho que ele veio a falecer esse ano, que é meu tio, ele não é meu tio, mas eu considero ele como se fosse um tio pra mim*” (Alex).

A qualidade dos vínculos sociais tem uma dimensão central na estruturação das redes sociais porque os laços estabelecidos entre os sujeitos podem significar tanto proteção quanto limitação da mobilidade social. Assim, se por um lado a predominância de laços fortes em uma comunidade é fundamental para a coesão social, contribuindo para gerar pertencimento, identificação e fortalecimento das relações identitárias, por outro lado, é um obstáculo para romper com a reprodução das condições de pobreza. É o que afirma Mark Granovetter ^18^, na medida em que as redes sociais muito coesas e densas tendem a ser mais “redundantes”, isto é, compostas por indivíduos dotados de atributos sociais similares. A “redundância” dos vínculos restringe a circulação de bens, de pessoas e de informações, de maneira que os laços fracos - dada a menor necessidade de fidelidade - favorecem a maior extensão e diversidade das interações sociais. Fala-se, assim, da “fortaleza dos laços fracos”, mais eficaz na proteção dos indivíduos por propiciar circuitos de reciprocidade que lhes permitem acessar recursos para superar situações de vulnerabilidade. O que explica, por exemplo, o fato de as redes sociais verificadas em comunidades de baixa renda de países latino-americanos serem significativamente caracterizadas pela presença de laços fortes e territorializados, ou com grande tendência ao localismo - concentradas na região de moradia ^5^^,^^6^.

**Figura 1:**
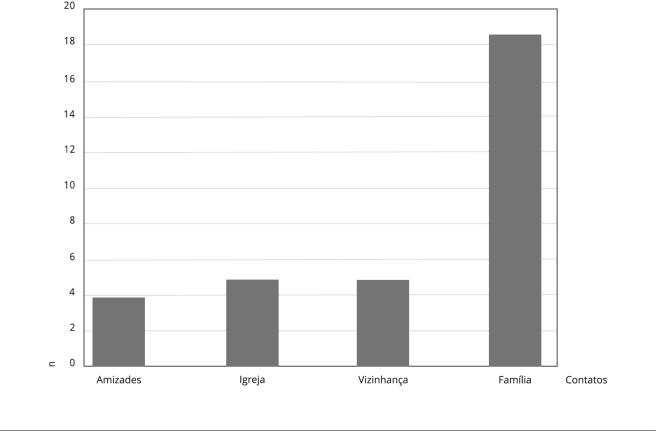
Intensidade dos vínculos mais fortes.

Na comunidade estudada, a intensidade dos vínculos foi explorada perguntando aos entrevistados em qual rede eles têm os vínculos mais fortes (Figura 1).

**Figura 2:**
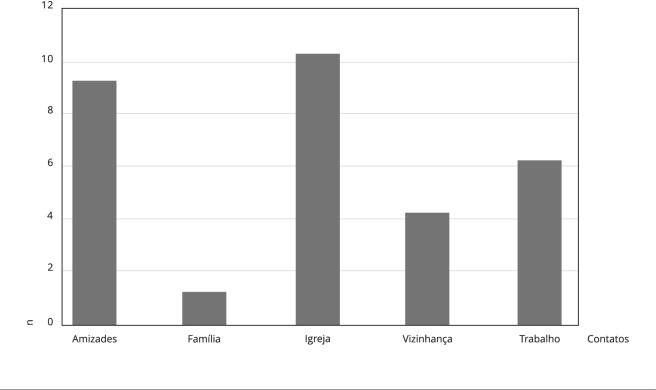
Concentração de nós por esfera social.

Os entrevistados podiam citar mais de uma rede, por isso o número de respostas ultrapassa o total de entrevistas. Como se vê, na percepção dos entrevistados, as redes familiares são as que concentram os laços mais fortes. Antes disso, eles foram questionados sobre onde se localiza a maior parte de seus contatos, se dentro ou fora do bairro e/ou da Regional Alterosas, além de em quais redes se situam esses contatos (Figura 2).

Embora tenha havido um equilíbrio nas respostas à primeira pergunta - metade respondeu ter mais contatos no bairro e/ou na Regional Alterosas e metade, fora -, quando se questionou em quais redes se situam esses contatos (Figura 2), as respostas evidenciaram o contrário: do total de menções às esferas sociais em que se concentra a maioria dos contatos, 22 correspondem às redes endógenas, isto é, dentro do bairro ou, no máximo, da Regional Alterosas, o que aponta para seu caráter mais local, confirmando o perfil descrito pela literatura. Outro aspecto importante de ser observado nas redes dos entrevistados é que a percepção da intensidade dos vínculos não necessariamente se relaciona com as redes em que se concentra o maior número de contatos, nem mesmo com a frequência dos encontros, mas com as relações de confiança, mais especificamente com a expectativa de poder contar com as pessoas em caso de necessidade. Por isso, a família foi mais citada como a esfera que abriga os laços mais fortes, ainda que não corresponda àquela mais acessada para a obtenção de recursos, como será visto adiante.

A tendência ao localismo das redes também reflete na duração dos vínculos na trajetória dos entrevistados. Amizades feitas no bairro, sobretudo entre os moradores antigos, tendem a se manter mesmo com a mudança para outros locais. As amizades feitas na escola tendem a se manter entre as redes formadas durante o ensino fundamental, quando cursado em escola do bairro ou da Regional Alterosas. As redes constituídas durante o ensino médio e cursos técnicos tendem a se romper, talvez porque as escolas pertençam a outras localidades, principalmente na região central de Betim. As redes formadas em universidades não se mostraram muito relevantes, o que se justifica, de acordo com os entrevistados, pela fase da vida em que esses vínculos são constituídos, com os integrantes estando já casados, com família constituída ou muito focados na vida profissional.

Outro aspecto analisado foi a utilização das redes sociais para a mobilização de recursos, ou seja, o tipo de capital social presente nessas relações, suas características e sua distribuição, a partir das redes mais acessadas para obtê-lo. Com isso, é possível identificar os padrões de sociabilidade mais ou menos funcionais à alocação de estoques de capital social no território, de acordo com a natureza dos vínculos em cada esfera social. Compreende-se o conceito de capital social como o “*conjunto de recursos atuais ou potenciais que estão ligados à posse de uma rede durável de relações mais ou menos institucionalizadas de interconhecimento e de inter-reconhecimento ou, em outros termos, à vinculação a um grupo* (...)”, nos termos de Pierre Bourdieu ^19^ (p. 67). O capital social não é uma propriedade individual, mas se situa no vínculo entre as pessoas. Dessa maneira, as redes de sociabilidade a que o indivíduo pertence modelam sua estrutura de oportunidades porque o capital social consiste tanto nas relações sociais, que permitem aos indivíduos reivindicar o acesso aos recursos dos integrantes das redes, quanto na qualidade desses recursos ^20^^,^^21^.

A Tabela 1 apresenta o tipo de capital social acessado em cada rede pelos entrevistados.

**Tabela 1:** Concentração de capital social por esfera social.

Recursos	Rede social				
	Família	Vizinhança	Trabalho	Amizades	Igreja
Vaga de emprego	11	3	2	10	-
Apoio emocional	10	3	4	8	9
Ajuda financeira	9	9	-	-	-
Ajuda com moradia	4	-	-	-	-
Cuidados de saúde	4	12	-	-	-
Alimentos	-	10	-	-	7
Cuidado de crianças/Parentes	-	8	-	-	-
Obra/Construção	-	3	-	-	-
Vigiar casa	-	7	-	-	-
Empréstimo de utensílios	-	7	-	-	-
Informações/Orientações	-	3	-	4	6
Formação/Qualificação	-	-	-	6	4

Confirmando o caráter local das redes, as mais utilizadas pelos entrevistados para a aquisição de recursos são as da vizinhança e as da família, respectivamente. Como dito anteriormente, apesar de as redes familiares serem apontadas como as de laços mais fortes, é na vizinhança (sobretudo os vizinhos antigos) que se concentra a maior parte do capital social acessado pelos entrevistados. Ressalta-se que esses dados se referem ao tipo e às características do capital social acessado em cada esfera, e não ao seu volume, que não é possível apreender por técnicas qualitativas. O volume de capital social da comunidade será medido na próxima etapa da pesquisa por meio de técnica específica.

A vizinhança é a esfera social em que se concentram os seguintes tipos de capital social, por ordem de importância: (1) cuidados de saúde; (2) alimentos; (3) recursos financeiros; (4) cuidado de crianças e parentes; (5) vigiar a casa em momentos de ausência, empréstimo de utensílios; (6) informação/indicação para vaga de emprego, apoio emocional, ajuda em obras (construção e pequenos reparos), auxílio com informações e orientações diversas. Já a família é a esfera acessada para: (1) informação/indicação para vaga de emprego; (2) apoio emocional; (3) ajuda financeira; (4) ajuda com moradia (empréstimo ou doação), auxílio com informações e orientações diversas. Ainda nas redes primárias, as amizades são acionadas pela maioria dos entrevistados para: (1) informação/indicação para vaga de emprego; (2) apoio emocional (conversas, conselhos, acolhimento); (3) formação e qualificação (informação sobre cursos e ajuda nos estudos); (4) auxílio com informações e orientações diversas.

Com relação às redes secundárias, os vínculos profissionais não se mostraram muito fortes nas entrevistas. As interações, geralmente, se limitam ao ambiente de trabalho e, quando ultrapassam o espaço profissional, se centram mais em encontros ocasionais. Essas redes são mais acessadas para provisão de (1) apoio emocional e (2) informação/indicação para vaga de emprego. De maneira bem menos expressiva, foram citadas as instituições de ensino como esferas sociais para auxílio em cursos de formação e qualificação na trajetória de alguns entrevistados, em especial a concessão de bolsas de estudo. Por outro lado, grande parte do capital social presente nas redes de sociabilidade secundária dos entrevistados advém dos grupos religiosos que frequentam: (1) apoio emocional; (2) alimentos; (3) auxílio em informações e orientações diversas; (4) auxílio na formação/qualificação (vaga em escola católica, bolsa de estudos em escola particular de ensino formal e de língua estrangeira), esse último bem menos expressivo e proveniente de igrejas fora da região de moradia - redes exógenas. Por sinal, os entrevistados que tiveram as melhores oportunidades de formação educacional/profissional em sua trajetória, com exceção dos oriundos da classe média, tiveram ajuda de algum contato de uma rede externa, como a patroa da mãe, que é empregada doméstica, ou de igrejas.

Como era de se esperar em razão do localismo das redes, o capital social do grupo de entrevistados está fortemente concentrado nas redes de sociabilidade primária (vizinhança, família e amizades), o que remete à discussão de Granovetter ^18^ sobre a “redundância” das redes constituídas por laços fortes. E, mesmo quando alocado nas redes de sociabilidade secundária, o capital social se concentra na região de moradia, isto é, nas igrejas locais. Até mesmo o fato de a família aparecer como esfera de maior provisão de informação e indicação de vagas de emprego, por ser uma rede constituída por laços fortes, dá pouca margem para o acesso a recursos que possibilitem mobilidade e ascensão profissional. Ou seja, as esferas sociais em que há maior concentração de capital social dos entrevistados evidencia o elevado grau de homofilia de suas redes, caracterizadas pela maior presença de pessoas com os mesmos atributos sociais do ego.

Em contrapartida, o tipo de capital social presente nas redes estudadas, voltado para a provisão de recursos básicos e imediatos (práticas assistenciais), para a prestação de serviços e para o apoio social, é um importante fator de proteção social para as comunidades. Esse tipo de capital é fundamental para fomentar o círculo de reciprocidade, aumentando a capacidade potencial para a ajuda mútua ^6^^,^^20^. Além disso, possibilita ganhos coletivos, como a produção de solidariedades locais, o fortalecimento dos laços comunitários, a formação de vínculos afetivos e identitários, bem como a estruturação de pactos e práticas coletivas, elementos essenciais para a mobilização social em prol de ações que visem ao bem comum ^22^^,^^23^.

No que se refere às redes secundárias ou associativas, as igrejas se destacaram como principal espaço de sociabilidade. Quando solicitados a falar sobre sua participação em grupos e associações, a maioria (25) relatou suas experiências em comunidades religiosas. É a esfera social que reúne a vizinhança, a família, as amizades, sendo frequentada como missão (realização de tarefas e atividades semanalmente), mas também como lazer (por meio de atividades organizadas pelos membros, como feiras, barraquinhas, festas), além dos próprios rituais (missas e cultos). As organizações religiosas têm centralidade junto a essas comunidades, por meio de obras sociais, provisão de recursos e suporte religioso. Por se localizarem, em sua maioria, no bairro onde os indivíduos moram, as redes religiosas ajudam a fortalecer (intensificar) os vínculos de vizinhança e de amizade porque concentram e reúnem os moradores nas suas atividades. Nota-se que quando os moradores frequentam a mesma igreja, os vínculos de vizinhança parecem ser ainda mais fortes.

Ao contrário do esperado, a participação em grupos da igreja católica predominou entre os entrevistados, assim como sua presença e atuação nos bairros. Dado o elevado número de templos evangélicos na Regional Alterosas e, como afirma a literatura, uma crescente adesão das periferias ao neopentecostalismo ^20^, esperava-se que as igrejas evangélicas tivessem maior centralidade nesses bairros, tanto na produção de solidariedade entre os moradores como na importância e atuação dessas instituições na região. Quando se perguntou aos entrevistados sobre as instituições e organizações de maior atuação nos bairros ou que poderiam contribuir para o desenvolvimento de algum projeto de políticas públicas para a comunidade, a maioria mencionou alguma instituição ligada à igreja católica.

Outra rede secundária que apareceu nas entrevistas, porém bem menos significativa (4), foi a dos grupos esportivos, destacando os times de vôlei e os grupos de corrida/caminhada. A participação política e o ativismo são pouco significativos entre os entrevistados, a maioria nunca participou de movimentos sociais, partidos políticos, associações, entre outros. Em Betim, é muito comum a participação na política se dar em forma de apoio a candidatos durante as eleições. A política formal, por sinal, foi bastante criticada. Há uma indignação com os representantes e com a maneira como eles, segundo os entrevistados, se aproveitam de questões sociais e comunitárias para benefício próprio. Há um descrédito na política formal, e é recorrente a fala de que projetos comunitários teriam apoio das comunidades se não estivessem atrelados a políticos.

## Considerações finais

Corroborando as hipóteses, as redes sociais dos entrevistados se mostraram bastante localistas (com a maior parte dos vínculos concentrados no bairro de moradia ou, no máximo, se estendendo para a Regional Alterosas), muito voltadas para a sociabilidade primária (família e vizinhança) e com baixa tendência ao associativismo (partidos políticos, associações de bairro, movimentos sociais), sendo a sociabilidade secundária voltada, predominantemente, para a participação em grupos religiosos. No que se refere à intensidade dos vínculos, as entrevistas confirmam o que é discutido pela literatura, isto é, a tendência muito comum em bairros de periferias urbanas de haver laços fortes entre os vizinhos, que se traduzem em um esquema de ajuda mútua (reciprocidade). Na trajetória dos entrevistados, a vizinhança se mostrou como importante rede de reciprocidade e de provisão de recursos no cotidiano, dada a proximidade física e a duração das relações.

A análise das redes sociais do território evidencia alguns aspectos importantes para a elaboração de novas estratégias de mobilização social no âmbito da proposta de vigilância em saúde a ser implementada no município. Embora as redes sociais caracterizadas pela presença de laços fortes não favoreçam o acesso às oportunidades e aos bens sociais, dificultando a mobilidade social, esse tipo de sociabilidade é um importante fator de integração social e de acúmulo de capital social comunitário. Com o apoio social representando o principal mecanismo de solidariedade, essas redes estimulam as trocas, a reciprocidade, a cooperação e a interdependência dos membros de uma comunidade. Por isso, elas são um importante instrumento para a implementação de políticas públicas, na medida em que, quando mobilizadas, se tornam uma estratégia de reforço do tecido social e, consequentemente, de ampliação da autonomia, da participação social e da cidadania. As redes religiosas, inclusive, com presença significativa no cotidiano dos moradores, mesmo sendo do tipo associativo (secundárias), se mostraram fortemente ancoradas na prestação de “ajuda” social, pautada por valores solidários. Essas redes, como trata a literatura, operam como um fator de proteção social para as classes populares ^24^.

Assim, dada a centralidade dessas redes, concentrando grande parte do capital social presente no território, é possível utilizá-las como canais de mediação ou pontes (*brokers*) entre as ações dos comitês populares e o restante da comunidade, ampliando o alcance da proposta de vigilância em saúde. A compreensão da estrutura das redes permite identificar aquelas mais propícias para o desenvolvimento de ações coletivas, especialmente quando seus padrões de sociabilidade estão intrinsecamente associados à territorialidade, com base na proximidade espacial e social ^6^. Ou seja, é o ambiente social adequado para estimular a participação voltada para assuntos locais, desde que essas redes sejam instrumentalizadas para que consigam se organizar e desenvolver autonomia para intervir e propor soluções para os territórios. Uma das estratégias seria a criação de “relés sociais”, conectando as redes preexistentes a outras, externas ao território, dando origem a novas redes ^7^. Como as redes locais são muito homogêneas ou “redundantes”, nos termos de Granovetter ^18^, os “relés sociais” possibilitam a entrada de novos recursos não acessados por seus integrantes: conhecimento técnico, científico, político, habilidades, compartilhamento de experiências. Essas novas redes, agora constituídas por laços fracos, passam a acessar o capital social que permite mobilizar os recursos da esfera pública, processo essencial para a estruturação de ações coletivas ^6^.

Desse modo, os comitês populares podem operar como redes de mediação entre as redes preexistentes no território e outras externas, já que elas não se conectam de forma espontânea, mas a partir da elaboração de políticas sociais que estimulem a formação de capital social nas esferas de sociabilidade primária ^9^. Por fim, acredita-se que os comitês populares também possam estimular essas redes, sobretudo as religiosas, a utilizarem seu repertório cultural e simbólico para trabalhar questões de interesse dos bairros, como promoção da saúde, construção de territórios saudáveis e sustentáveis, desenvolvimento local, geração de renda, melhoria da infraestrutura e preservação ambiental.
